# Bio-Based Surfactants and Biosurfactants: An Overview and Main Characteristics

**DOI:** 10.3390/molecules30040863

**Published:** 2025-02-13

**Authors:** Gloria Romero Vega, Paola Gallo Stampino

**Affiliations:** Department of Chemistry, Materials and Chemical Engineering “G. Natta”, Politecnico di Milano, Piazza Leonardo da Vinci 32, 20133 Milan, Italy; gloria.romero@polimi.it

**Keywords:** bio-based surfactants, biosurfactants, agro-residue substrates

## Abstract

Natural surfactants are surface-active molecules synthesized from renewable resources (i.e., plants, animals, or microorganisms) and possess properties comparable to conventional surfactants, making them an environmentally friendly potential alternative to petrochemical surfactants. Additionally, they exhibit biological properties such as anti-microbial properties, biodegradability, and less toxicity, allowing their use in everyday products with minimal risk to human health and the environment. Based on their mode of production, natural surfactants can be classified into first-generation or bio-based surfactants and second-generation or biosurfactants, although their definition may vary depending on the author in the literature. This review offers an extensive classification of bio-based surfactants and biosurfactants, focusing on their composition, natural sources, production methods, and potential applications across various industries. Furthermore, the main challenges and future perspectives are discussed.

## 1. Introduction

Although petrochemicals have been widely utilized across numerous industrial sectors for an extended period, their negative impact on the environment and the health of living organisms is well-documented [1,2]. Most conventional products are entirely or partially derived from fossil resources, and their production processes are highly standardized [3,4]. However, these products pose significant challenges in terms of disposal, as many chemicals are not readily degradable, leading to their accumulation in soil and water sources for hundreds of years. Moreover, their degradation products may be toxic to living organisms [2,5,6,7].

In recent years, sustainability has become a global priority, encompassing economic, environmental, and social dimensions, known as the “3Ps”—people, planet, and profits [8]. From an environmental perspective, sustainability aims to mitigate the ecological and climatic crises that destabilize the planet [5]. Consequently, the development of bio-based products utilizing compounds derived from natural and renewable resources, such as biomass, represents a more sustainable pathway with the potential to significantly reduce CO_2_ emissions [5,9].

One of the most prevalent petrochemical-derived compounds in daily life are surfactants. These molecules, known for their ability to reduce surface or interfacial tension between two liquids, are widely employed in the detergents, cosmetics, food, and pharmaceutical industries [1,7,10]. Their amphiphilic structure enables functionalities such as emulsification, wetting, foaming, suspension, and lubrication [5,11]. Surfactants are categorized based on the ionic charge of their hydrophilic polar head into non-ionic, anionic, cationic, and zwitterionic (or amphoteric) types [3,10]. However, conventional surfactants are known for their toxicity and resistance to degradation, which exacerbates the environmental impact and bioaccumulation concerns [12]. Indeed, their consumption grew during the COVID-19 pandemic, due to heightened cleaning and sanitization protocols aimed at minimizing viral spread [13]. Moreover, due to their high solubility in water, residual surfactant contents often persist after wastewater treatments and are subsequently discharged into aquatic ecosystems [2]. Furthermore, these compounds can impair the efficacy of microorganisms used in wastewater treatment processes due to their anti-microbial properties [2].

To address these challenges, the surfactant industry is innovating more sustainable alternatives, synthesizing surfactants from renewable resources such as plants, animals, and microorganisms [1,5]. In the literature, these alternatives are referred to as natural surfactants, renewable surfactants, green surfactants, bio-based surfactants, or biosurfactants, though the definitions may vary depending on the source [1,3,6,7]. For the purposes of this paper, the term “natural surfactant” encompasses all surfactants derived from renewable sources.

**Figure 1:**
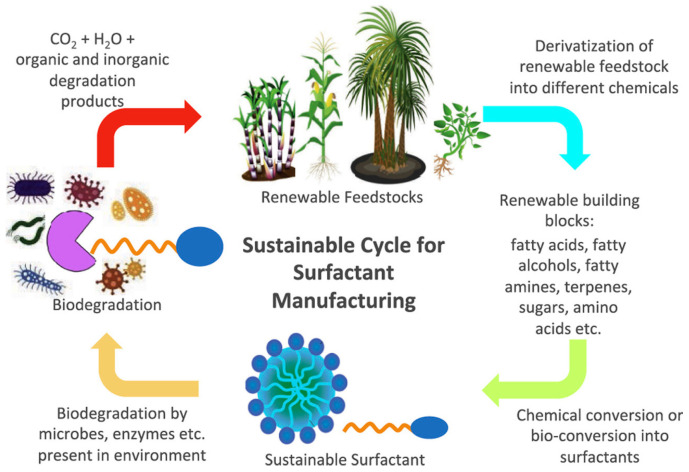
Ideal sustainable cycle for developing new generations of natural surfactants, taken from [3]. Copyright 2020 Elsevier Ltd.

In addition, natural surfactants exhibit properties comparable to their petrol chemical counterparts, with added benefits such as biodegradability, biocompatibility, lower toxicity, stability under extreme conditions, and eco-friendliness. These features result in reduced environmental impact due to the use of renewable feedstocks that lower CO_2_ emissions during production (Figure 1) [6,14,15].

Although petrochemical-based surfactants continue to dominate the market, the share of natural surfactants has been steadily increasing, now accounting for 40% of the total surfactant market. The bio-based surfactant and biosurfactant market is estimated to grow from USD 19 billion in 2023 to USD 26 billion in 2032 [16]. Although there is an optimistic view regarding the market diffusion and use of bio-based surfactants, it is worth noting that there are currently many issues related to the costs of large-scale production, separation processes, batch-to-batch variability, and potential toxicity/allergic reactions.

This review provides an extensive classification of natural surfactants based on their synthesis methods, the compound generation, and the nature of the renewable feedstocks used. The aim is to offer a comprehensive understanding of these surfactants, highlighting their diverse properties and contemporary applications, and to promote research into methods to address scalability and performance issues.

## 2. Surfactants

Petrol-based surfactants, also known as tension-active agents, are amphiphilic molecules consisting of a polar hydrophilic region (head) chemically bonded to a hydrophobic tail, typically composed of a linear, branched, or aromatic hydrocarbon chain [12,17]. Based on the ionic charge of the hydrophilic head, surfactants are classified into four main categories: non-ionic, anionic, cationic, or zwitterionic/amphoteric [3,10].

Due to their structure, these molecules can reduce the surface tension between two liquid phases (typically 20–72 mN/m) [3]. Their performance is influenced by two critical parameters: the hydrophilic–lipophilic balance (HLB) and the critical micelle concentration (CMC) [5]. The HLB value is determined by the balance between the hydrophilic and hydrophobic regions of the molecule, and it is associated with the specific functions of surfactants (Table 1) [5,17].

The HLB number is usually calculated using the Griffin method:

(1) $$\mathrm{HLB}=20\cdot \frac{X}{W},$$

where X and W are the molecular mass of the hydrophilic region of the surfactant and the molecular mass of the whole molecule, respectively [18]. A high HLB value indicates dominance of the hydrophilic region, while a low HLB value represents lipophilic dominance [17,19].

Even though HLB is commonly used for surfactant formulations, the hydrophilic–lipophilic difference (HLD) method overcomes its drawbacks by including the effects of oil and surfactant nature, temperature, salinity, and ionic strength to predict the macro properties of surfactants with greater accuracy [20,21,22]. This semi-empirical method relates experimental parameters to describe the affinity difference in surfactants for the water and oil phases [22].

The general HLD equation is described as follows:

(2) HLD=+Cc−k·EACN−αΔT+f(S),

where Cc is the surfactant-specific characteristic curvature, EACN is the equivalent alkane carbon number, ΔT is the difference between the actual and the reference temperatures, f(S) is the salinity function (i.e., ln(S) for ionic surfactants and b·S for non-ioninc surfactants), and α, b, and k are empirical constants related to the surfactant [21,22,23,24]. Most surfactants with more hydrophilic behaviors have a very high value of Cc, favoring a positive HLD [23].

**Figure 2:**
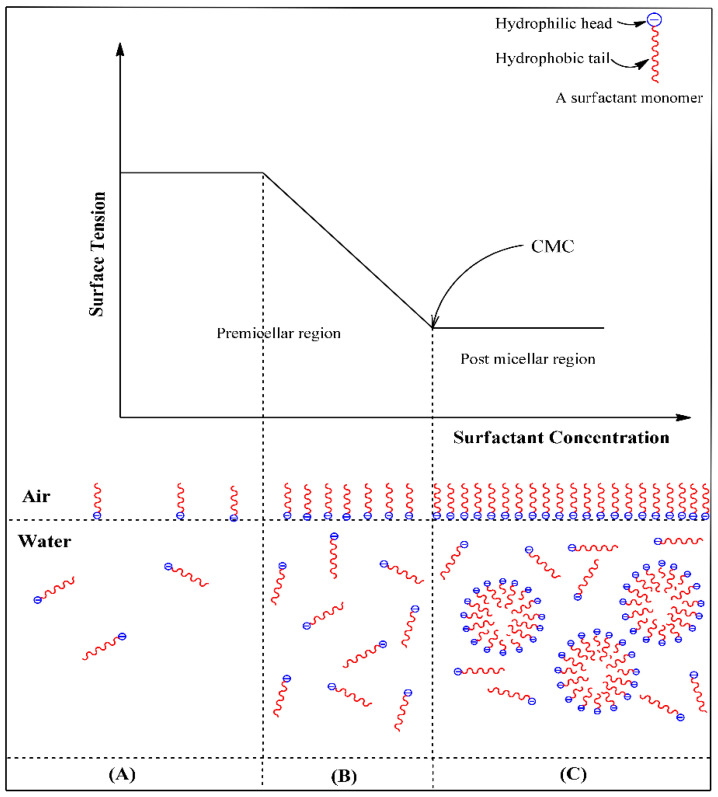
Representation of micellization behavior of surfactants in aqueous solution. (**A**) There is less reduction in surface tension at low surfactant concentrations. (**B**) Increasing the surfactant concentration, surface tension reduces steadily until CMC is reached. (**C**) No more changes in surface tension are verified beyond CMC. Taken from [25]. Copyright 2021 by the authors.

The CMC is the concentration value at which surfactants self-assemble into micelles in the presence of polar or non-polar solvents, reaching the lowest stable surface tension value [3,5,25]. Once micelles are formed, surface tension is no longer affected [25]. Above the CMC value, surfactants reduce surface tension and form micelles of various shapes—ranging from spherical to more complex structures—that influence their dynamic and rheological properties and the overall functional performance [2]. Furthermore, the CMC values are temperature-dependent; higher temperatures lead to lower CMC values (Figure 2) [26].

## 3. Effects on Environment and Health

**Figure 3:**
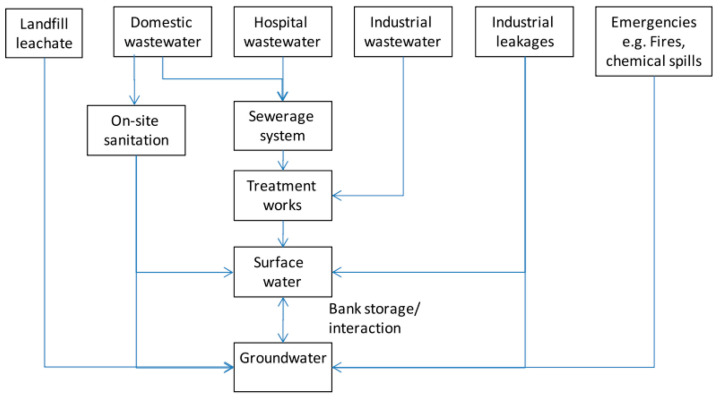
Different pathways for contaminants to reach groundwater in the urban environment, taken from [2]. Copyright 2020 Elsevier B.V.

Surfactants derived from non-renewable sources can cause several forms of damage to the ecosystem, being toxic to living beings, non-biocompatible, not easily degradable, and their degradation may produce harmful by-products [6,7,27]. Water bodies are particularly affected by residues from detergents and other contaminants present in domestic and industrial wastewater (Figure 3). These pollutants contribute to the death of aquatic species, human health issues, reduced oxygen levels in the water, disruption of the water cycle, and overall ecosystem degradation [2,5,27,28]. The concentration of surfactants in rivers varies seasonally, with lower levels observed in winter due to increased water flow and higher levels in summer [2,5].

Due to the molecular properties of surfactants, wastewater treatments are insufficient to remove all the surfactants from water sources. Approximately 60% of surfactants remain untreated, necessitating the disposal of part of the water. Surfactants also reduce the efficiency of the microorganisms used for pollutant removal and contribute to increased bacterial resistance due to their constant exposure [2].

Some effects on living beings are [2,28]:

- Aquatic life: Reduction in the mucus layer of fish, leading to higher mortality rates and genetic deformities in some species;
- Humans: Toxicity and irritation of the skin, eyes, and respiratory system;
- Plants: Decreased photochemical energy conversion efficiency and toxicity for certain crops, such as lettuce;
- Marine plants: Reduction in sunlight availability caused by foam accumulation;
- Marine microorganisms: Suppression of bacterial population.

To address these issues, various international regulatory organizations aim to mitigate the negative impacts of surfactants, including the FDA (Food and Drug Administration) in the USA and the European Chemical Agency (ECHA) in Europe [29].

## 4. Natural Surfactants

The depletion of fossil resources and growing sustainability awareness have encouraged the surfactant market to explore natural and renewable sources. These alternatives aim to minimize environmental harm and enhance product benefits [1,5]. Natural surfactants are surface-active molecules produced from renewable materials including plants, animals, microorganisms, or agricultural by-products and waste [1,5]. Often referred to as bio-based surfactants, green surfactants, or biosurfactants, their definitions vary according to the literature. For instance,

- Bio-based surfactants: produced by chemical synthesis, from renewable resources.
- Biosurfactants: biosynthesized by living organisms, such as microorganisms [10,15].

For consistency, this paper will use the term “natural surfactant” to encompass all surfactants derived from different renewable sources.

Natural surfactants have an amphiphilic structure, similar to fossil-based surfactants but with more variability in the division of the hydrophilic and hydrophobic regions [30,31]. Furthermore, their CMC is lower than the CMC of conventional surfactants, allowing the use of significantly smaller concentrations. For plant-based surfactants, the CMC value depends on factors like plant type and extraction method (Table 2) [25].

**Figure 4:**
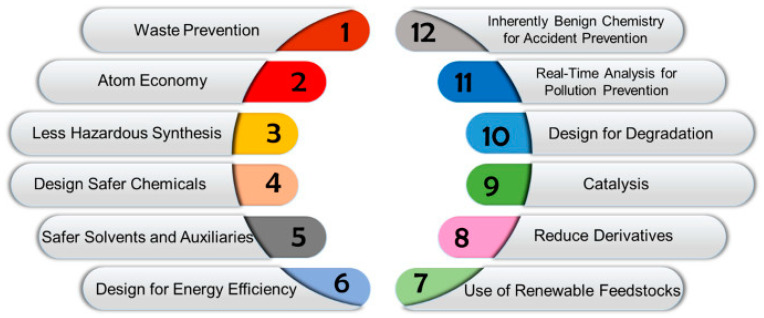
The twelve principles of green chemistry, taken from [17]. Copyright 2022, The Authors.

In addition to standard surfactant functions like emulsification, lubrication, and wetting, natural surfactants may also exhibit anti-microbial, anti-viral, anti-cancer, and anti-corrosive properties. They are highly resistant to temperature, salinity, and pH variation [2,6,7,38,39]. Due to these properties, they are highly used in food, cosmetics, pharmaceuticals, textile–leather sectors, and in metallurgical–petrochemical industries, in which they are used for applications such as ion flotation, soil remediation, the bioremediation of the marine environment, the treatment of oily effluents, and metal removal [2,7,40,41]. Furthermore, natural surfactants fulfill many of the principles of green chemistry, due to the raw material used as substrate and their degradability, reusability, low environmental toxicity, wide availability, and biocompatibility (Figure 4) [6,17,38,39].

Renewable surfactants can be classified based on their ionic charge, renewable resources origin, or the methods and processes used for their synthesis. According to the compound generation, natural surfactants can be classified as [5,30,42]:

- First-generation natural surfactants or bio-based surfactants are extracted and purified or chemically synthesized from plant-based and animal-based feedstock to achieve the desired surfactant structure, such as saponins and alkyl polyglucosides (APGs).
- Second-generation natural surfactants are biosynthesized directly by plants, animals, or microorganisms through biological processes, such as fermentation, using renewable raw materials, by-products, or agro-industrial waste. Known as biosurfactants, sophorolipids and rhamnolipids are the most commercially recognized in this category.

### 4.1. First-Generation Natural Surfactants: Bio-Based Surfactants

Most bio-based surfactants are derived from plants and animals. The hydrophilic component typically consists of carbohydrates, glycerol, and amino acids, while the hydrophobic part is made up of fatty acids obtained from different plants or waste cooking oils [3,17]. Both components can be chemically or enzymatically linked together to form the amphiphilic structure of surfactants [12].

In Table 3 are listed some of the most well-known bio-based surfactants nowadays:

#### 4.1.1. Glycerol-Based Surfactants

Crude glycerol, a by-product of the biodiesel and oligo-chemical industries, has become an important renewable resource. Its quality depends on production and purification processes [58,62,63]. Crude glycerol can be derived from various chemical processes such as saponification and hydrolysis reactions in oleochemical plants, transesterification in biodiesel production, or fermentation with yeast, bacteria, or algae [58,63,64].

Due to its low cost and availability, glycerol is a sustainable hydrophilic head group used for natural surfactant production. Glycerol-based surfactants can be synthesized through direct esterification of glycerol and fatty acids or the transesterification of glycerol natural fats/oils or other fatty acids [3,65]. These surfactants exhibit properties such as emulsification, alkali tolerance, foam stability, and solubilization of hydrophobic dyes in aqueous medium, making them ideal for laundry applications [54,55,56,57,58].

#### 4.1.2. Animal-Based Surfactants

Nowadays, animal-derived surfactants are primarily used in biomedical fields, in particular for treating Respiratory Distress Syndrome (RDS), a major cause of respiratory failure in newborns. The most commonly FDA-approved lung surfactants are derived from porcine or bovine lung tissue, which is purified and extracted using organic solvents [66,67,68,69]:

- Poractant alfa (Curosurf): contains a high concentration of porcine-derived phospholipids;
- Calfactant (Infasurf) and beractant (Survanta): derive from bovine species.

Recent studies suggest that animal-derived surfactants could be utilized as drug carriers for pulmonary therapeutics and other treatments due to their rapid spreading properties [70]. Furthermore, the use of exogenous surfactants, especially those from animals’ derivation, has been shown to significantly reduce morbidity and mortality in clinical applications [69].

#### 4.1.3. Plant-Based Surfactants—Saponins

Plants are a major source of natural surfactants, with saponins serving as non-ionic bio-based surfactants naturally produced by more than 100 species of vascular plants and some marine organisms. Saponins act as chemical barriers against pathogens and herbivores [1,25]. They are found in various plant parts, including leaves, roots, flowers, seed pericarp, and fruits [25,53]. They could be extracted through conventional methods (i.e., maceration, soxhlet extraction, and reflux extraction) or advanced extraction techniques (i.e., ultrasound-assisted extraction, microwave-assisted extraction, enzyme-assisted extraction, supercritical fluid extraction, pressurized liquid extraction, and accelerated solvent extraction) [25,52].

**Figure 5:**
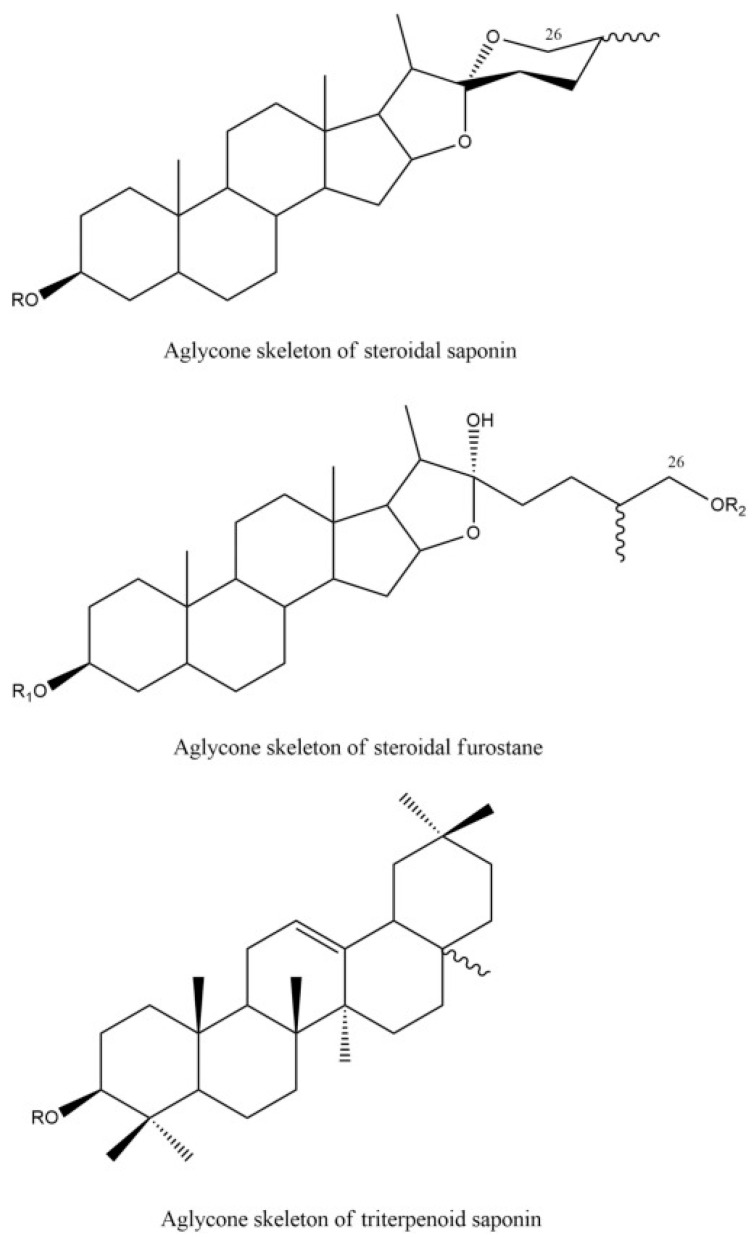
Chemical structure of steroidal saponins, steroidal furostane, and triterpene saponins, taken from [71]. Copyright 2023 Elsevier B.V.

Their concentration and composition depend on the plant type, plant part, growth conditions, and extraction method [25,53]. Saponins consist of non-polar aglycones linked to sugar molecules [1,25]. According to their aglycone type, saponins can be classified into two classes (Figure 5):

- Triterpene saponins with a pentacyclic nucleus composed of 30 carbon atoms;
- Steroidal saponins are composed of a nucleus of 27 carbon atoms [1,51]. There is another steroidal skeleton, called the furostane skeleton, in which the pentacyclic aglycone structure is maintained due to the glycosidic connection involved in the hydroxyl group at the 26-position of the fresh plant material [71].

While according to the number of sugar chains, saponins are classified as mono-, di-, or tri-desmosidic saponins [1,25]. The most common sugars present in saponins are mostly composed of D-glucose, D-galactose, L-arabinose, L-rhamnose, D-xylose, and D-fructose [1,25,30].

Due to their structural diversity, saponins have an extensive list of applications in different sectors as emulsifiers, foaming agents, detergents, shampoos, solubilizers, insect repellents, food additives, cosmetics, wetting agents, pharmaceuticals, drug carriers, antioxidant, anti-diabetic, anti-obesity, anti-fungal, anti-microbial, anti-inflammatory, anti-tumoral, analgesic, molluscicides, remediation, among other functions [1,25,30,41,51,52,53].

Table 4 shows some saponin sources and their utilization:

#### 4.1.4. Sugar-Based Surfactants

Carbohydrates derived from industrial waste and agricultural biomasses (i.e., sugar and starch) are used as hydrophilic head groups in bio-based surfactant production due to their low cost, availability, and versatile properties [17,81,82]. Sugar-based surfactants are characterized by good biocompatibility, high biodegradability, environmental compatibility, good surface activity, low toxicity, dermal tolerability, and high wetting, foaming, and emulsifying properties [3,81,82,83]. They are commonly found in pharmaceuticals, detergents, cosmetics, food, and many personal care products [46,81,84]. The most commercialized sugar-based surfactants are alkyl polyglucosides (APGs), sucrose esters, and sorbitan esters [83].

##### Alkyl Polyglucosides (APGs)

**Figure 6:**
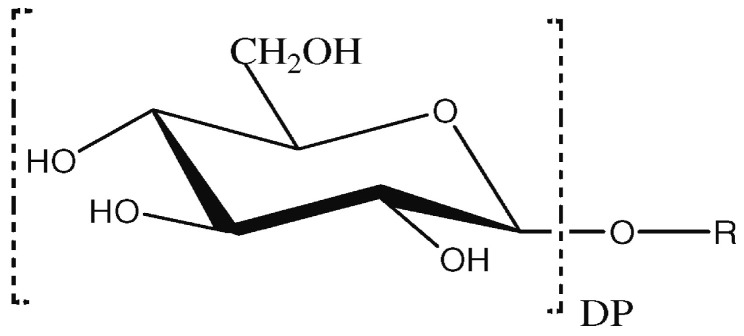
Chemical structure of alkyl polyglucoside (APG), taken from [85]. Copyright 2013, The Authors.

APGs are non-ionic sugar-based surfactants widely used across industries due to their biodegradability, biocompatibility, and comparable properties with their counterparts [17]. They are synthesized through trans-acetylation or acetylation process using glucose (i.e., from corn or wheat) and fatty alcohols (i.e., from palm kernel or coconut oil) [17,19]. The hydrophobic and hydrophilic part of APG is clearly separated, and the hydrophilic region of the molecule is usually composed of one to five condensed glucoside parts (DP = 1–5) (Figure 6) [85].

APGs exhibit excellent properties, including thermal stability, emulsification, foaming and wetting properties, anti-bacterial activity, thickening effects, dermatological compatibility, and ocular safety, with a high electrolyte tolerance. Due to all these properties, APGs are ideal for detergents, cosmetics, pharmaceuticals (i.e., drug carrier delivery), personal care products (i.e., shampoos, shower gels), petrochemical industries (i.e., enhancing oil recovery) and food industries [43,44,45,46,47,48,49,50].

##### Sucrose Esters

Sucrose is a low-cost organic compound synthesized by most plants as an early product of photosynthesis [61]. In the surfactant industries, sucrose derived from sugarcane is used as a hydrophilic head group to produce non-ionic sucrose esters, in which sucrose is linked via an ester bond to a fatty acyl tail from coconut oil [17,86,87]. Sucrose esters can be synthesized through the esterification of triglycerides or the transesterification of the fatty acid methyl ester with sucrose in the presence of a basic catalyst [17,61].

Sucrose esters are commonly applied as emulsifiers in cosmetics, cleansing agents, and personal care products. Additionally, they have potential as drug permeability enhancers due to their biocompatible and eco-friendly properties [59,60].

##### Sorbitan Esters

Sorbitan esters, known commercially as Spans, are non-ionic surfactants consisting of a hydrophilic head group made of sorbitol—a polyol rich in hydroxyl groups found in many fruits, seaweed, and plants—and a hydrophobic tail group composed of fatty acids of various origins (i.e., vegetable oils or animal fats) [82,88,89]. They are widely used as emulsifiers in the food, cosmetics, and pharmaceutical industries. Their properties, including hydrophilic–lipophilic balance (HLB) values, vary on the degree of esterification and the nature of the fatty acid [82,88].

In the literature, sorbitan esters have also been used for the fabrication of stable oleo-foams, adsorption on copper and cuprous oxide surfaces, the production of composite oleo gels, and as nanoparticles for a novel topical ocular drug delivery system [88,90,91,92].

### 4.2. Second-Generation Surfactants: Biosurfactants

**Figure 7:**
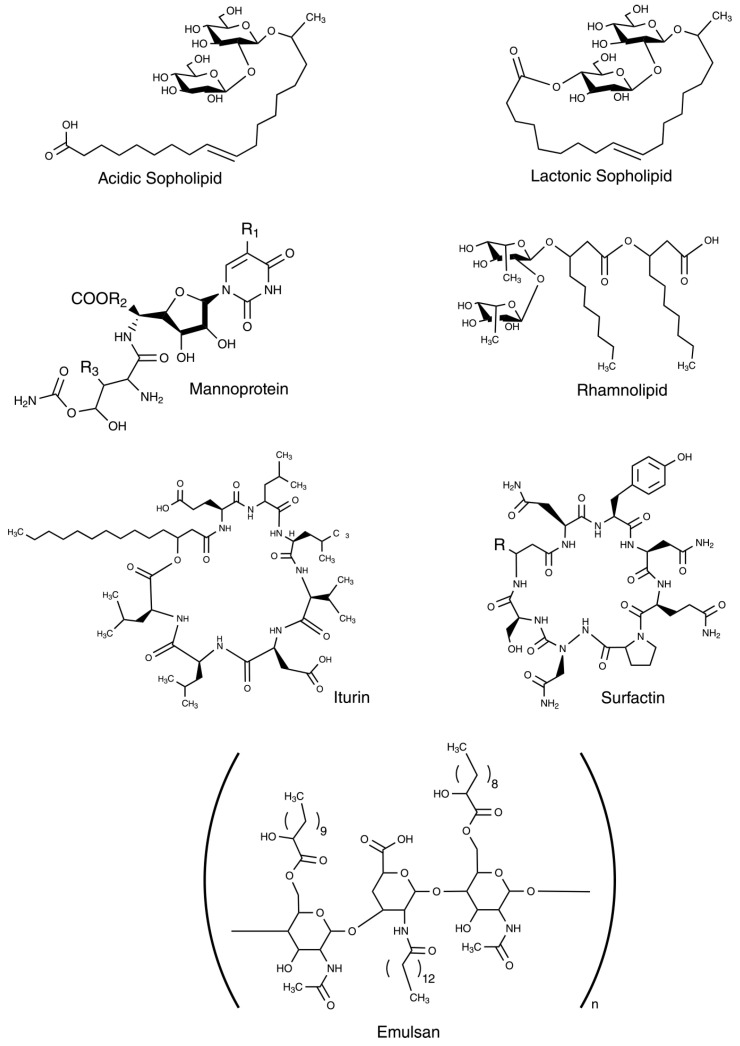
Different types of biosurfactants, taken from [5]. Copyright 2022, Sociedade Brasileira de Química.

Biosurfactants, also referred to as microbial surfactants are secondary metabolites produced through enzymatic reactions by microorganisms (bacteria, yeasts, and filamentous fungi) during the late exponential phase of growth. These reactions utilize renewable resources as substrates (i.e., carbohydrates and vegetable oils) [29,93,94,95]. These natural surface-active molecules are involved in many cellular communication processes and can either be secreted extracellularly or remain associated with cell surfaces [29,94]. On an industrial scale, biosurfactant production is sustainable, with its efficiency greatly enhanced by advancements in biotechnology [12,95]. Their complex chemical structures and various properties are influenced by the producing microorganisms, the type of substrates used, medium composition, and culture condition (Figure 7) [29,93].

The main classes of biosurfactants, according to their molecular structure, are glycolipids, lipopeptides, proteins, lipoproteins, phospholipids, polymeric biosurfactants, polysaccharides, lipopolysaccharides, and fatty acids [1,6,96,97]. Another classification divides them by molecular weight:

- Low molecular weight (<1200 g/mol): glycolipids, lipopeptides, and phospholipids, which are more effective at reducing surface tension;
- High molecular weight (>45,000 g/mol): polysaccharides, proteins, lipoproteins, and lipopolysaccharides, highly used as bio-emulsifiers [1,6,94,98].

Most biosurfactants have a non-ionic or anionic nature and find applications in different fields, including food, agriculture, cosmetics, pharmaceuticals, environmental protection processes (i.e., oil recovery, bioremediation, and oil mobilization), personal care, and cleaning products [1,93,94,95].

Compared to chemical surfactants, biosurfactants offer numerous advantages, such as lower critical micelle concentration (CMC), higher biodegradability, reduced toxicity, biological activities (i.e., anti-fungal, anti-viral, anti-bacterial, and anti-cancer properties), resistance to extreme pH and temperature variation, enhanced ecological compatibility, etc., [94,96,99,100].

The most well-known commercialized microbial surfactants are glycolipids, such as sophorolipids, rhamnolipids, and mannosylerythritol lipids (MELs) [95]. Table 5 summarizes some of the biosurfactants by category.

#### 4.2.1. Glycolipids

Glycolipids are amphiphilic compounds that are naturally present in living organisms, where they play an important role in many biological processes, such as molecular recognition, cell adhesion, interaction with cellular membranes, and signal transduction, among other biological functions [95,138]. On an industrial scale, they are produced by fermentation processes; some examples of glycolipid biosurfactants that are already commercialized are sophorolipids, rhamnolipids, and mannosylerythritol lipids (MELs) [95]. They are composed of a carbohydrate group linked through glycosidic bonds to lipid groups. They are derived from bacterial species like *Pseudomonas* sp. and *Bacillus* sp., or yeast such as *Candida bombicola* [94,139]. These biosurfactants are applied in the cosmetic and detergent industries as emulsifiers, solubilizers, wetting agents, foaming, dispersants, and penetration enhancers [95,99]. In petrochemical fields, they are widely used for enhanced oil recovery [99]. Moreover, glycolipids exhibit anti-cancer, anti-bacterial, and anti-viral activities [138].

##### Rhamnolipids

Rhamnolipids are anionic biosurfactants primarily produced by a bacteria called *Pseudomonas aeruginosa*. They are among the most extensively studied biosurfactants due to their efficient production, short incubation periods, and easy microorganism cultivation [101,102,140]. Their structure is determined by the composition of the rhamnose moiety (polar) and lipid moiety (apolar), as well as the carbon chain length (ranging from C8 to C24). The rhamnose moiety (polar part) consists of one or two di-L-rhamnose units, while the lipid moiety (apolar part) consists of saturated or unsaturated (mono- or polyunsaturated) β-hydroxy fatty acid chains [94,101]. They are commonly produced via fermentation processes (i.e., submerged or solid-state fermentation) and separated through processes such as centrifugation or sedimentation [94]. They are considered low or non-toxic biosurfactants, able to degrade with properties such as anti-adhesive, anti-bacterial, anti-viral, anti-tumor, and emulsifying activities, making them applicable across industries including agriculture, medicine, food, cosmetics, detergents, biotechnological, and bioremediation applications [94,101,102]. Additionally, rhamnolipids are used in applications such as:

- Transportation enhancers in nano-remediation;
- Degreaser formulation;
- Pesticides removal from contaminated soils;
- Ion collectors in ion flotation;
- Production of lipid-based antimicrobial nanomaterials;
- Bioremediation of marine oil spills;
- Natural nanocarriers of photosensitizers in photodynamic therapy for targeting abnormal cells and microorganisms [141,142,143,144,145,146,147].

##### Sophorolipids

Sophorolipids are one of the most commercialized glycolipid biosurfactants due to their high productivity and stability on an industrial scale [106,148]. These compounds are produced through the fermentation of saccharides and hydrophobic carbon sources by several yeast species, *Starmerella bombicola* being the most well-studied species and most efficient sophorolipid producer [93,104,148]. Additionally, other renewable substrates derived from waste materials are increasingly utilized for their production, including [104]:

- 52% agricultural waste, such as oils, sugarcane bagasse, potato scraps, corn straw, rice husk, corn cob, etc.;
- 18% animal fat-derived waste;
- 30% service sector bio-waste, such as restaurants and catering waste oils, food companies’ by-products, and supermarket leftovers.

Furthermore, crude glycerol, a by-product of biodiesel production, is an optimal substrate for sophorolipid synthesis [104].

Sophorolipids are constituted of a hydrophilic carbohydrate headgroup (2-*O*-β-d-glucopyranosyl-d-glucopyranose) linked to a hydrophobic alkyl chain (C16–C18) by a glycosidic binding between the anomeric C atom of the sugar and the hydroxyl group of the fatty acid [93,104]. These biosurfactants excel at reducing water surface or interfacial tension from 72 to 30–40 mN/m at a critical CMC of 40–100 mg/L [33,105]. They are also applied as lubricants, solubilizers, detergents, foaming agents, emulsifiers, wound healing and anti-cancer effects, and anti-microbial agents against several bacteria, viruses, and fungi species, among other applications in different sectors such as cosmetics, food, bioremediation, agriculture, pharmaceutical, and medical industries [103,104,105,106]. According to the esterification of the hydroxylated fatty acid, sophorolipids can be divided into lactonic and acidic types [33]. Lactonic sophorolipids exhibit anti-bacterial, spermicidal, anti-tumor, and surface tension-lowering activities but are poorly soluble in water. Instead, acidic sophorolipids enhance solubility and foam production, and are highly used in bioremediation and the detergent, food, and cosmetic industries [93,104,105].

In the literature, sophorolipids are being used in the synthesis of anti-microbial bioactive films for strawberries and control of human skin pathogens, MEOR (microbial-enhanced oil recovery), the formulation of nano-emulsions to control pathogenic bacteria, and the phytoremediation of heavy metal contaminated soil, etc., [33,149,150,151].

##### Mannosylerythritol Lipids (MELs)

**Figure 8:**
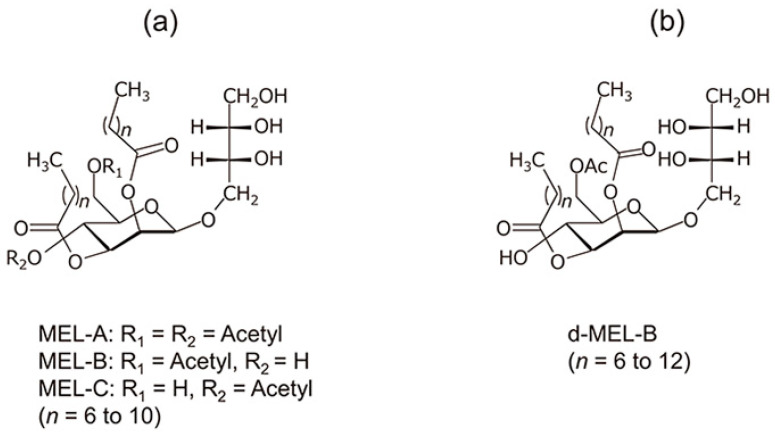
Chemical structures of mannosylerythritol lipids (MELs) produced by different yeasts from the *Pseudozyma* family. (**a**) MEL-A, MEL-B and MEL-C produced by *P. antarctica*; (**b**) diastereomer of MEL-B (d-MEL-B) produced by *P. tsukubaensis*, taken from [95]. Copyright 2022 by Japan Oil Chemists’ Society.

Mannosylerythritol lipids (MELs) are non-ionic microbial biosurfactants and consist of a 4-*O*-β-d-mannopyranosyl-d-erythritol polar head group and hydrophobic tails comprising fatty acids and/or acetyl groups [95,108,152]. They are commonly synthesized by fungi species such as *Moesziomyces* and *Ustilago* sp. or by yeast strains belonging to the *Pseudozyma* family (Figure 8) [95,152]. The chemical structure, fermentation process, and culture medium composition significantly influence MELs’ biological properties [107]. The most common substrate used for MELs production is vegetable oils (i.e., olive, rapeseed, and soybean oils), often combined with glucose to maximize microbial growth and biosurfactant yield [152].

Due to their biological properties, MELs have been used as anti-tumoral, anti-biofilm, and anti-bacterial agents, emulsifiers to induce cell differentiation, in enzyme activation/inhibition, gene transfection, and gene therapy in biomedical applications [95,97,107,108,109,110]. In the cosmetic industry, instead, they are used due to their antioxidant and protective properties in skin cells, moisturizing effect for dry skin, and their potential as anti-melanogenic properties in skincare products [95,107,110].

#### 4.2.2. Lipopeptides

Another type of bacterial biosurfactant is lipopeptides that are biosynthesized by several bacterial microorganisms such as the *Bacillus*, *Pseudomonas*, *Paenibacillus*, *Streptomyces*, and *Enterobacter* species through multienzyme complexes called non-ribosomal peptide synthetases (NRPSs) [98,153,154,155].

The most common chemical structure of a lipopeptide is a lipophilic fatty acid chain (12 to 19 carbon atoms), linked together through an amide or ester bond with a hydrophilic peptide ring composed of 7–10 amino acids [155,156,157].

There are three important lipopeptide families (surfactins, iturins, and fengycins), which differ in amino acid composition and fatty acid chain length [115,153,158]. These lipopeptide families are mainly produced by *Bacillus subtilis* bacteria [159].

These biosurfactants are applied in different sectors (i.e., chemical, agricultural, pharmaceutical, medicine, cosmetic, and food fields) due to their properties [156]. Some of their usual applications are emulsifiers, solubilizers, anti-microbial activity, biocontrol agents, anti-fungal activity, plant defense stimulants, anti-tumor agents, and anti-viral and anti-bacterial agents [98,115,124,156,160].

Recently, lipopeptide biosurfactants have been used against SARS-CoV-2 due to their anti-viral activities [161]. They also can induce mitochondrial apoptosis in pathogenic fungi and destabilize microbial membranes, contributing to their anti-microbial property [124,158]. Furthermore, lipopeptides inhibit synergistic interactions and enhance activities when different families are co-produced [155,157,158].

To lower production costs, agricultural residues such as sugarcane molasses, rice straw, cassava flour, and wheat straw, as well as oil substrates such as coconut, canola, sunflower, or olive oil, are commonly used as feedstocks. Other waste-derived substrates, including animal fat, starch, and vegetable juice, also support economical lipopeptide production (i.e., animal fat, molasses, starch, corn steep liquor, vegetable juice) [98,156].

##### Surfactin

Surfactin is a low-molecular-weight biosurfactant (1036 Da) composed of a cyclic heptapeptide (seven amino acid residues such as L-Glu1-L-Leu2-D-Leu3-L-Val4-L-Asp5-D-Leu6-L-Leu7) and a long β-hydroxy acid chain (12–16 C atoms) linked via a lactone bond. It is primarily produced by the *Bacillus* species through microbial fermentation or chemical synthesis [116,162,163,164,165]. This biosurfactant was first identified in 1968 in cultures of *Bacillus subtilis* strains [165,166].

Surfactin can be applied across multiple industries, including agriculture, cosmetics, food, medicine, and petroleum. It acts as an antibiotic for human and plant health and is considered the most effective biosurfactant in reducing surface tension at a low CMC. It can reduce surface tension from 72 to 27 mN/m at a CMC of 20 mg/L, enabling the use of low concentrations in practical applications [35,116,162,163,164]. This biosurfactant, at high concentrations, shows anti-bacterial effects but has fewer anti-fungal properties than other lipopeptide biosurfactants [115,124,158].

Some of the most common functions used in different sectors are shown in Table 6.

Additional applications include its use as drug delivery carriers in multiple sclerosis, as collectors for froth flotation of sulfide minerals, as organogelators for the production of oleogels, as coatings to facilitate the delivery of silver nanoparticles against drug-resistant bacterial biofilm infections, and as hyperglycemia alleviators in mice with type 2 diabetes [162,163,169,170,171].

##### Iturin

The iturin family (i.e., iturin A, C, D, and E) consists of cyclic lipopeptides made up of seven α-ammino acids (L-Asn-D-Tyr-D-Asn-L-Gln-L-Pro-D-Asn-L-Ser) and a β-ammino acid bond linked to long aliphatic chain (14–17 carbon atoms). These are connected via an ester bond, forming a cyclic structure. Iturin is extracellularly produced by *Bacillus* bacteria and closely related strains [117,118,121,159,172]. Together with surfacin, they are widely employed in biotechnological or biomedical fields due to their anti-bacterial, anti-fungal, anti-biofilm, anti-cancer, anti-viral, and hemolytic agents [117,118,119,120]. Other examples of applications include biocontrol agents in agriculture, microbial-enhanced oil recovery in the petroleum sector, and as emulsifiers and inhibitors of fat globule aggregation in food industries, etc., [117,121]. In the literature, iturin has also been used to form a complex with silver nanoparticles to enhance anti-fungal and anti-bacterial activities for wound healing [173].

##### Fengycin

The fengycin biosurfactant family is composed of ten amino acids (8 amino acids form a lactone ring) and a β-hydroxy fatty acid, forming a ring structure with a side chain length of 14–21 carbon atoms. They are produced by the *Bacillus* species, which typically simultaneously secrete multiple fengycin members during fermentation, complicating isolation and purification [158,174]. They exhibit anti-fungal, anti-microbial, anti-tumor, and anti-viral properties, making them useful for inhibiting mold in food preservation [122,158,174,175]. They also show great potential as biocontrol agents in agriculture to reduce the use of pesticides [124,176]. Furthermore, fengycin is a natural antibiotic used in many industries, such as medicine, food, and agriculture. Other applications are environmental and as a biological control in aquaculture [123]. Moreover, the presence of sugar supplementation and the addition of external precursors is important to increase the level of essential amino acids for their production and the condition of the medium [123,175,177].

#### 4.2.3. Surface-Active Proteins—Hydrophobins

Hydrophobins are surface-active globular proteins with low molecular weight (<20 kDa) produced by filamentous fungi, such as *Penicillium*, *Aspergillus*, *Trichoderma*, extremophilic species, or mycorrhizal fungi., which are involved in their growth and development phases [4,128,129]. They have β-barrel structures (similar to each other) and are composed of 100–150 amino acids with a conservative part of eight cysteine residues, forming four intracellular disulfide bonds [4,128,129,178].

**Figure 9:**
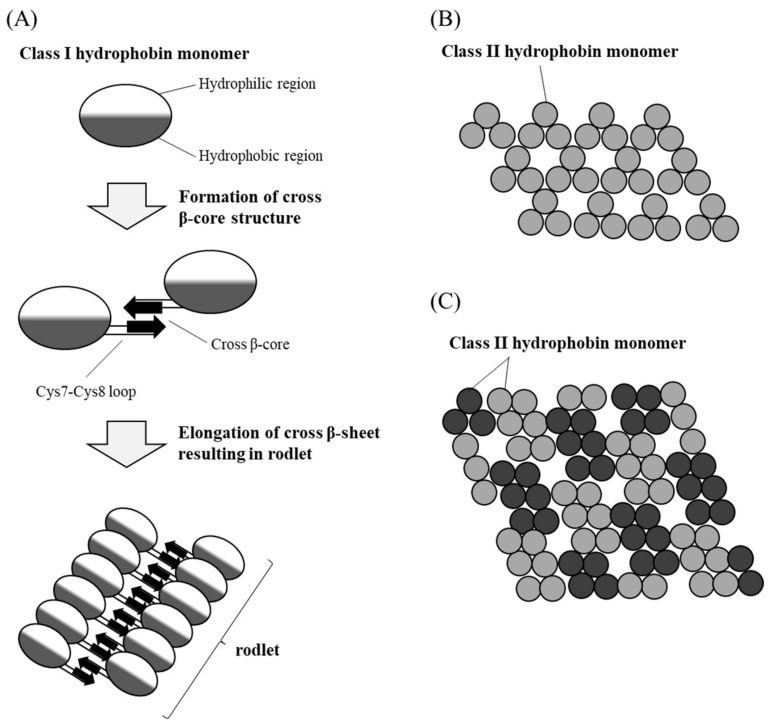
Hydrophobin self-assembly schematic representation. (**A**) Class I hydrophobin forms rod-like insoluble polymers (rodlets). (**B**,**C**) The self-assembled structure of class II hydrophobin is less robust and composed of (**B**) 3- or (**C**) 6-molecule units, taken from [129]. Copyright 2022, The authors.

Hydrophobins are classified according to their assembly characteristics at hydrophobic–hydrophilic interfaces, amino acid sequences, solubility, and hydropathy patterns (Figure 9) [128,129,179]:

- Class I: Form rod-like insoluble polymers extracted from ascomycetes and basidiomycetes fungi [129,180]. They can form fibrillar layers (β-sheet conformation), similar to amyloid fibrils [4,181];
- Class II: Found in ascomycetes (i.e., *Trichoderma*, *Fusarium*, and *Neurospora*) but not in the *Aspergillus* species. They do not have a rod-like structure like Class I, but have a cysteine conservative region [129,180]. In contrast with Class I hydrophobins, Class II hydrophobins are less robust, forming regular crystalline structures with a random spiral conformation [4,181].

Those hydrophobins with intermediate properties are considered Class III (produced by *Aspergilli*) or pseudo-class I (produced by *Trichoderma*) [129,181]. Moreover, the production of hydrophobins depends strongly on many factors, such as the type of filamentous fungi, culture medium, environmental conditions (nutrition and light), the growth stage of filamentous fungi, and cellular localization, which affect the expression profiles of multiple hydrophobins genes [129,180].

In the literature, hydrophobins have been studied for biomineralization applications, modification of the wettability of solid surfaces such as Teflon, as an immune-suppressive barrier in drug formulations, hydrophobic drug solubilization and delivery in biomedical applications, anti-microbial coating for biomaterials, food dispersion, protein purification process, biosensors, foam, and emulsion stabilizers, among other applications [4,127,128,129,130]. It was also demonstrated that they can enhance PET hydrolysis in the presence of a potential biocatalyst called PETase [182].

Different homologous and heterologous methods are applied to enhance the native expression of hydrophobins. For example, homologous methods are those in which genetic engineering techniques are involved to increase the biosurfactant yield. In contrast, heterologous methods utilize plants or plant cell cultures for hydrophobin production [181].

#### 4.2.4. Polymeric Biosurfactants

Polymeric biosurfactants are high-molecular-weight active-agent molecules, with Alasan and Emusal being among the most well-known examples, which are both generated by *Acinetobacter* species [183]:

- Alasan is an anionic heteropolysaccharide and protein complex containing proteins rich in alanine (45 kDa), produced by *Acinetobacter radioresistens.* It is widely used due to its emulsifying, solubilizing, and surface activities properties [131,132,183,184].
- Emulsan is an anionic lipo-heteropolysaccharide and protein complex (1000 kDa). Its hydrophilic carbohydrate backbone is linked to a hydrophobic fatty acid moiety through *O*-ester or *N*-acyl linkages. It is produced by *Acinetobacter calcoaceticus* RAG-1 and *Acinetobacter calcoaceticus* BD4 bacteria. This biosurfactant is an excellent emulsion stabilizer at low concentrations, making it a promising candidate for protein and drug delivery [131,132,133,183,184].

Another polymeric biosurfactant is Liposan, which is a heteropolysaccharide–protein complex produced by *Candida lipolytica*. It comprises 83% carbohydrates and 17% proteins, and it is widely used as an emulsifier and solubilizer in the cosmetic and food industries owing to its exceptional emulsion-stabilizing properties [100,132,134].

## 5. Main Challenges and Future Perspectives

Although bio-based and biosurfactants have huge advantages compared to petrol-chemical ones, they show difficulties in industrial scale-up and commercialization [5].

Some of the main drawbacks are [5,10,29,31,100]:

- High cost of production: Their complex production includes steps such as fermentation and purification processes or enzymatic routes that are still expensive. In addition, the selected substrate cost could increase the price of the final product.
- Low productivity: The fermentation and purification steps increase the production time. Furthermore, the purification of natural surfactants consists of several steps that produce large amounts of waste, decreasing production recovery.
- High sensibility to environmental factors: Their complex chemical structure and various properties are significantly affected by environmental factors such as temperature, salinity, and pH.
- Variability in production yield: Due to their high environmental sensibility, the production batch of natural surfactants varies due to the type of substrates used, environmental conditions during cultivation, the substrate extraction method, the fermentation method, etc. For biosurfactants, the medium composition, the culture condition, and the producing microorganisms also affect the final product.
- Lack of standardization: Most natural surfactants are still at the laboratory scale.

To overcome high production costs, it is important to consider using natural by-products as natural substrates. By-products from agriculture, the food industry, and other sectors in which their wastes come from natural resources could help to reduce the cost of the final product, not considering the previous steps for obtaining the substrate, such as the extraction method. Furthermore, natural by-product substrates do not compete with human and animal feed [8,185]. Also, the advances in genetic engineering and the development of novel strains for the production of biosurfactants could enhance the biosurfactant production yield, reducing production costs [186].

These challenges also open up many opportunities in the research field, such as the creation of more sustainable production methods for natural surfactants, the increase in knowledge and the value of natural substrates that can be used for their production, the reduction in lack of standardization to compete with conventional surfactants, and the discovery of other applications in different sectors due to their varied properties.

## 6. Conclusions

The search for new natural substrates for the production of various everyday products is not only an important topic for the scientific community, but also for industries aiming to mitigate risks for living beings and the environment. Natural surfactants play a significant role due to their renewable origins and huge properties, including biological functions (i.e., anti-microbial and anti-tumor agents), which broaden their application across multiple sectors. However, their production cost is higher compared to conventional surfactants primarily due to the sensitivity of natural substrates to the environmental conditions during plant cultivation or microbial culture, extraction, and purification processes, leading to variability in biosurfactant (or bio-based surfactant) yields. Moreover, most natural surfactants are currently produced at a laboratory scale, and their production is not standardized like petrol-based surfactants, reducing their commercial appeal in the global market. Despite this, the valorization of bio-waste from different industries, growing environmental concerns worldwide, and the continuous development of new production technologies are helping to bridge the gaps associated with biosurfactant limitations, making them a safer choice for the formulation of new products. Furthermore, the adoption of green chemistry principles by industries and research into the environmental impacts of different natural surfactants are advancing the industrial-scale production of more sustainable natural surfactants.

## Figures and Tables

**Table 1:** HLB: hydrophilic–hydrophobic balance, taken from [5]. Copyright 2022 Sociedade Brasileira de Química.

HLB Range	Applications
4–6	Water/oil emulsifier
7–9	Wetting agent
8–18	Oil/water emulsifier
13–15	Detergent
15–18	Solubilizer

**Table 2:** CMC and surface tension of some non-renewable and natural surfactants.

Type	Name	CMC [g/L]	Reduced Surface Tension [mN/m]	Temperature [°C]	References
Non-renewable surfactants	Sodium lauryl sulfate (SLS)	2.004	39.2	20	[25]
	Sodium dodecyl sulfate (SDS)	2.36	25	N.A.	[2]
	Cetyl trimethyl ammonium bromide (CTAB)	1.131	N.A.	25	[25]
		353	33.4	25	
Bio-based surfactants	Decyl glycoside from D-glucose (APG)	0.994	26	N.A.	[32]
	Decyl glycoside from D-xylose (APG)	0.301	28	N.A.	[32]
	Betula pendula saponins (leaves)	0.24	45.7	20	[25]
	Bellis perennis saponins (flowers)	0.076	36.8	20	[25]
	Genipa americana saponins (fruits)	0.65	31.39 ± 0.15	25 ± 1	[25]
Biosurfactants	Sophorolipids	0.04–0.1	30–40	N.A.	[33]
	Rhamnolipids	0.01–0.02	26	N.A.	[2]
	Hydrophilic mannosylerythritol lipid (MEL)	0.1	27	25 ± 2	[34]
	Hydrophilic mannosylerythritol lipid (MEL)—G	0.125	30.5	25 ± 2	[34]
	Surfactin	0.02	27	N.A.	[35]
	Iturin C_3_	0.04183	N.A.	20	[36]
	Hydrophobin	0.005	30	N.A.	[37]

**Table 3:** The most well-known bio-based surfactants.

Name	Derived/Produced from	Function	References
Alkyl polyglucosides (APGs)	Synthesized through the trans-acetylation or acetylation process between glucose (i.e., from corn or wheat) and fatty alcohols (i.e., from palm kernel or coconut oil).	High tolerance of electrolytes, thermal stability, emulsifying, foaming, and wetting properties, biodegradability, anti-bacterial activity, thickening effects, dermatological compatibility, and ocular safety.	[17,19,43,44,45,46,47,48,49,50]
Saponins	Found as a blend of various saponin types in diverse parts of the plants.	Emulsifiers, foaming agents, detergents, shampoos, solubilizers, insect repellents, food additives, cosmetics, wetting agents, pharmaceuticals, drug carriers, antioxidants, anti-diabetic, anti-obesity, anti-fungal, anti-microbial, anti-inflammatory, anti-tumoral, analgesic, molluscicides, remediation, among other functions.	[1,25,30,41,51,52,53]
Glycerol-based surfactant	Glycerol and fatty acids through esterification or transesterification.	Emulsifying and solubilizing properties, alkali tolerance, foam stability, and laundry performances.	[17,54,55,56,57,58]
Sucrose sorbitans	Synthesized by esterification of triglycerides or transesterification of fatty acid methyl ester with sucrose in basic catalyst presence.	Emulsifiers in cosmetics, cleansing, and personal care products but could have potential as drug permeability enhancers due to their biocompatible and eco-friendly behaviors.	[17,59,60,61]

**Table 4:** Saponin applications in different sectors.

Saponin Source	Sectors	Applications	References
Yucca schidigera saponin	N.A.	Emulsifying and foaming agents, anti-inflammatory, anti-fungal, anti-parasitic, anti-tumoral, antioxidant, and anti-microbial activity.	[72]
Plants from northern Thailand such as litsea glutinosa, sapindus rarak, and acacia concinna.	Detergent, personal care, and medicine.	Detergents, shampoo ingredients, cleansing purpose such as spiritual cleansing during Thai new year. Traditional medicines due to their biological activities such as anti-spasmodic, anti-bacterial, and cardiovascular activities, etc.	[53]
Steroic saponins from Allii Macrostemonis Bulbus, a traditional Chinese medicine	Pharmaceutics	Anti-coagulation, lipid-lowering, anti-tumor, and antioxidant effects. Moderate anti-inflammatory effects on endothelial cells, anti-atherosclerotic effect.	[73]
Total saponins from Panax japonicus	Pharmaceutics	Anti-angiogenic effect in rheumatoid arthritis. Commonly used to alleviate the pathological symptoms of the digestive system, cardiovascular system, and central nervous system. Inhibitory effects on immune inflammation and general inflammation, bidirectional regulatory effects on immune function, and can restore the abnormal immune system. Regulated the intestinal microbiota to alleviate lipid metabolism disorders in aging mice.	[74,75,76,77,78]
Boron + saponins	Agriculture	An effective treatment in mitigating salinity stress for sweet potatoes, improving various growth parameters and physiological aspects.	[79]
Tea saponins, found in Camellia plants	Soil remediation	Desorb heavy metals from contaminated soil as well as enhancing their bioavailability. Improve the accumulation.	[80]

**Table 5:** Microbial biosurfactants classification.

Type	Name	Biosynthesized by	Applications	References
Glycolipids	Rhamnolipids	*Pseudomonas aeruginosa*	Anti-adhesive, anti-bacterial, anti-viral, anti-tumor, dispersing, emulsification, wetting, detergency, and de-emulsification activities, among other effects.	[94,101,102]
	Sophorolipids	*Starmerella bombicola* and other yeast	Lubricants, solubilizers, detergents, foaming agents, emulsifiers, wound healing and anti-cancer effects, and anti-microbial agents against several bacteria, viruses, and fungi species.	[103,104,105,106]
	Mannosylerythritol lipids (MELs)	Fungi species such as *Moesziomyces* and *Ustilago* sp., or yeast strains belonging to the *Pseudozyma* family.	Anti-tumoral, anti-biofilm, and anti-bacterial agents, emulsifiers, enzyme activation/inhibition, gene transfection and gene therapy in biomedical applications. Antioxidant and protective properties in skin cells, moisturizing effect for dry skin, and their potential as anti-melanogenic properties in skincare products.	[95,97,107,108,109,110]
	Cellobiose lipids	*Ustilago maydis*, *Ustilagomycetic* yeasts of the genus *Pseudozyma* such as *P. fusiformata*, *P. aphidis*, and *P. hubeiensis*.	Anti-microbial potential, phytopathogenic action against powdery mildew, yeasts and Gram-positive bacteria commonly associated with human infections. Additives for the formulation of colloids applied in the food and cosmetics industries, etc.	[4,111]
	Trehalose lipids	Gram-positive bacteria with high GC (Guanine and Cytosine) content: *Actinomycetales* such as *Mycobacterium*, *Nocardia*, *Corynebacterium*, and *Rhodococcus.*	Emulsifiers, wetting, foaming, solubilizers, anti-microbial, and anti-adhesive agents in biomedical, pharmaceutical, food, and environmental sectors.	[4,111,112]
	Xylolipids	Secreted by *Pichia caribbica* when grown in xylose-rich media. *Lactococcus lactis* LNH70.	Reduce the surface tension to 35.9 mN/m with a CMC of 1 mg/L, anti-bacterial activity against *S. aureus*. Antioxidant properties.	[4,113]
Glycolipid with polyol as polar moiety—polyol lipids	Liamocins	Produced by *Aureobasidium pullulans*, *Aureobasidium melanogenum*.	Anti-bacterial activity against strains of *Streptococcus* spp. Anti-microbial agent group, particularly in prophylactic applications. Inhibit the formation of oral biofilms of *S. mutans*, *S. sobrinus*, *and S. suis*, mainly by rupturing the pathogen’s cell membrane.	[4,114]
	Polyol esters of fatty acids (PEFA)	Secreted by genus *Rhodotorula* such as *Rhodotorula graminis* and *Rhodotorula glutinis*.	Anti-foam activities. Promote the formation of water-in-oil emulsions in water/octane mixtures. Promising prospects for therapeutic and environmental applications.	[4,114]
Lipopeptides	Surfactin	*Bacillus* species	Antibiotic properties for humans and plants. At high concentrations, they show anti-bacterial effects but has fewer anti-fungal properties than other lipopeptide biosurfactants.	[115,116]
	Iturin	*Bacillus* bacteria and closely related bacterial strains	Anti-bacterial, anti-fungal, anti-biofilm, anti-cancer, anti-viral, and hemolytic agents. Biocontrol agents in agriculture. Microbial-enhanced oil recovery in the petroleum sector. Emulsifiers and inhibitors of fat globule aggregation in food industries.	[117,118,119,120,121]
	Fengycin	*Bacillus* species	Anti-fungal, anti-microbial, anti-tumor, antibiotic, and anti-viral properties. Biocontrol agents.	[122,123,124]
	Viscosin	Synthesized by soil and marine bacteria such as *Pseudomonas* sp. (*Pseudomonas fluorescens*)	Anti-microbial effects against bacteria, fungi, protozoa, and human viruses. Involved in pore formation and destabilization of the cytoplasmic membrane of target cells.	[98,125]
	Lichenysin	*Bacillus licheniformis.*	Anti-microbial agent against important human pathogens. Pre-coating agents on various surfaces used in several indwelling medical devices and catheters in in vitro conditions.	[98,119,126]
Surface-active proteins	Hydrophobins	Filamentous fungi (i.e., *Penicillium*, *Aspergillus*, *Trichoderma*, extremophilic species, or mycorrhizal fungi).	Modification of wettability of solid surfaces (i.e., Teflon), immune-suppressive barrier, hydrophobic drug solubilization and delivery in biomedical applications, antimicrobial coating for biomaterials, food dispersion, protein purification process, biosensors, foam and emulsion stabilizers.	[4,127,128,129,130]
Polymeric biosurfactant	Alasan	*Acinetobacter radioresistens* bacteria.	Emulsifying, stabilizing, solubilizing, and surface activities.	[131,132]
	Emulsan	*Acinetobacter calcoaceticus* RAG-1 and *Acinetobacter calcoaceticus* BD4 bacteria.	Excellent emulsion stabilizer at low concentrations.	[132,133]
	Liposan	*Candida lipolytica*.	Emulsifying, solubilizing, and emulsion stabilizing properties.	[100,134]
	Mannanproteins	*Saccharomyces* spp. and *Kluyveromyces marxianus* of yeast.	Bio-emulsifiers, antioxidants, anti-bacterial and antibiotic properties, anti-tumor agents, and prebiotic components. It can act as a surfactant, reducing bacterial adhesion to the intestines and biofilm formation.	[135,136]
	Biodispersan	Generated by *Acinetobacter calcoaceticus* strains.	Emulsifying and stabilizing agents in the industry.	[137]

**Table 6:** Surfactin functions in different sectors.

Sector	Functions	References
Cosmetics	Anti-bacterial, emulsifying, washing, foaming, solubilizing, wetting, penetrating, dispersion, and low-toxicity characteristics.	[165,166]
Medicine	Anti-tumor, anti-virus and anti-inflammatory activities, anti-biofilm agent, immunosuppressive activity and maintenance of gastrointestinal homeostasis.	[124,162,164,166]
Agriculture	Biocontrol agents for plant diseases, systematic resistance in plants and promote plant growth and development. Formation of biofilm on plant roots.	[167,168]
Food	Additive for food processing and formulations due to their larvicidal, anti-adhesive and antimicrobial agents.	[169]

## Data Availability

No new data were created or analyzed in this study. Data sharing is not applicable to this article.
